# Assessing Parenting Interactions With Children: Spanish Validation of PICCOLO With Fathers

**DOI:** 10.3389/fpsyg.2021.747716

**Published:** 2021-10-15

**Authors:** Magda Rivero, Rosa Vilaseca, Fina Ferrer, Georgina Guilera

**Affiliations:** ^1^Department of Cognition, Development and Educational Psychology, Faculty of Psychology, University of Barcelona, Barcelona, Spain; ^2^Barcelona City Council, Barcelona, Spain; ^3^Department of Social Psychology and Quantitative Psychology, Faculty of Psychology, University of Barcelona, Barcelona, Spain

**Keywords:** parenting, positive parenting, fathering, child development, observational tools, PICCOLO

## Abstract

**Background/Objective:** To gain knowledge about mothers' and fathers' interactions with their sons and daughters, we need reliable and valid tools to assess parental behaviors that can be used for different caregivers and in a variety of cultural contexts. The aim of this study was to analyze the psychometric properties of Parenting Interactions with Children: Checklist of Observations Linked to Outcomes (PICCOLO) to assess fathers' interaction with their children at early ages. PICCOLO is an observational tool originally developed in the United States for mothers and fathers and previously validated in Spain with a sample of mothers.

**Methods:** One hundred and ninety-one father–child dyads were observed during free-play situations at home when the children were between 10 and 47 months of age (55.0% male). The fathers auto recorded 8–10 minutes of interaction and trained evaluators assessed the recordings with PICCOLO.

**Results:** Confirmatory factor analysis (CFA) confirmed the dimensional structure of the original version of the scale: four first-order factors (Affection, Responsiveness, Encouragement and Teaching) and one second-order factor (Parenting). The tool was found to have high inter-rater reliability at domain and total score level. Ordinal alpha and omega coefficients for each domain ranged between 0.79 and 0.85, and 0.64 and 0.79, respectively. No statistically significant differences were found in any PICCOLO domain or in the total score according to the child's gender. In assessments of the child's development with the Bayley-III scales, moderate positive correlations were found between Encouragement and receptive language (*r* = 0.32), and between Teaching and expressive (*r* = 0.34) and composite language (*r* = 0.31).

**Conclusion:** The Spanish version of PICCOLO can be used to assess fathers' parenting. As PICCOLO is clearly linked to intervention goals, it is of particular interest for practitioners in early intervention and family programs.

## Introduction

The increasing participation of women in the workforce and men in family life has led to rising interest in fatherhood in recent decades. A burgeoning body of literature has been focusing on questions such as the role of fathers in family life; the father's experience during pregnancy, childbirth, and childrearing; or father–child interactions and their contribution to the child's development (for a review, see Fitzgerald et al., 2020).

To gain knowledge about mothers' and fathers' interactions with their sons and daughters, we need reliable and valid tools to assess parental behaviors that can be used for different caregivers and in a variety of cultural contexts. We cannot assume that tools that have been validated with a mothers' sample can be directly used to assess fathers' behaviors.

The purpose of this study was to gather data on father–infant interaction using the Spanish version of Parenting Interactions with Children: Checklist of Observations Linked to Outcomes (PICCOLO), an observational measure of parental behaviors with young children (10–47 months) that was validated in Spain with mothers (Vilaseca et al., 2019b). The aim was to determine how fathers behave when they interact with their typically developing children. If the psychometric properties of PICCOLO in fathers were found to be poor, specific scales for fathers would need to be made.

### Fathers' Parenting and Child Development

It is well-established that the way mothers and fathers interact with their children at early ages in everyday life activities such as play, storytelling or other daily routines has an impact on the child's developmental outcomes. This is the case for typically developing children (Roggman et al., 2013a,b; Volling et al., 2019) and children with developmental delay or disabilities (Innocenti et al., 2013; Vilaseca et al., 2019a).

Literature that specifically analyzes father–child interactions at early ages has shown that some parental behaviors have been positively linked to the child's motor, social, cognitive, and linguistic developmental outcomes, as we will discuss below. We refer to all behaviors in adult–child dyadic interactions that promote the child's development as *parenting or positive parenting* (Roggman et al., 2013a,b).

Fathers' emotional availability, which refers to the overall affective quality of adult–child interactions, has been related to the child's emotional (Martins et al., 2016) and linguistic development (McMahon et al., 2019). Father–child attachment has also been linked to better socio-emotional, cognitive, and linguistic development of the child (Brown and Aytuglu, 2020). Some studies showed that fathers who interacted in a more sensitive, less intrusive way with their children, quickly and contingently responding to the child's behavioral and emotional signals and adjusting to his/her needs and interests, developed relationships that predicted attachment security (Brown et al., 2012; Fuertes et al., 2016; Bureau et al., 2020), promoted the child's linguistic development (Tamis-LeMonda et al., 2013) and his/her socioemotional competencies (Cabrera et al., 2017; Menashe-Grinberg and Atzaba-Poria, 2017). The father's support of the child's autonomy has been shown to predict the child's level of executive functioning during preschool years (Meuwissen and Carlson, 2015). Qualitative characteristics of the father's linguistic input to the child has also been linked to language development (Baker et al., 2015; Rowe et al., 2017; Reynolds et al., 2019).

Considering the family as a dynamic system, parenting behaviors are affected by, and affect, the parents' personality and personal characteristics, family relationships, work, or the child's development, among other variables (Fitzgerald et al., 2020). Accordingly, mothers' and fathers' parenting behaviors can present commonalities and differences and are complementary in their contribution to the child's development (Sameroff and Fiese, 2000; Cabrera et al., 2014; Vilaseca et al., 2020). If we assume that both mothers and fathers contribute to the child's development, as is well established in the literature (Fitzgerald et al., 2020), we need to have reliable, valid tools to assess maternal and paternal parenting.

### Tools to Assess Parenting

Parent reports are one of the main tools for assessing parenting. Some of the most internationally used parenting questionnaires are the Alabama Parenting Questionnaire (APQ) (Frick, 1991; Shelton et al., 1996) and the Baby Care Questionnaire (BCQ) (Winstanley and Gattis, 2013). The psychometric properties of the APQ were assessed with a sample of primary caregivers, 95% of whom were mothers. A recent validation of a short version of the APQ in Chile was also conducted with a mixed-gender sample with a very high proportion of mothers (87.1%). The factor structure, reliability, and validity of the Baby Care Questionnaire (BCQ) (Winstanley and Gattis, 2013) were analyzed using the same form for a mixed sample with 98% of mothers. After a review, we identified that mixed-gender samples with a very high proportion of mothers are very commonly used in analyses of the psychometric properties of parenting questionnaires. This is also the case of the Positive Parenting Scale (Gómez and Muñoz, 2014) in Chile or the Questionnaire for the Early Assessment of Parental Competencies in Spain (Vázquez et al., 2016).

Other parenting questionnaires, as the Parent–Child Relationship Inventory (PCRI) (Gerard, 1994) or the more recently created Comprehensive Early Childhood Parenting Questionnaire (CECPAQ) (Verhoeven et al., 2017) were administered to a more balanced sample. In the case of the PCRI, the sample included 55.2% of mothers, 39.1% of fathers and 5.7% of other primary caregivers, and the authors developed separate standards for mothers and fathers. The psychometric properties of the CECPAQ were analyzed according to the parents' sex, with a sample including 68.4% of mothers and 31.6% of fathers.

Some other tools include parent's reports collected by interview and some level of direct observation of parental behaviors during home visits, as is the case in the very broadly used Home Observation for Measurement of the Environment (HOME) (Caldwell and Bradley, 2001) and another similar tool developed in Spain (Velasco et al., 2014), the Etxadi-Gangoiti scale, which includes an interview, a questionnaire, and some direct observation of parental behaviors. The psychometric properties of the Etxadi-Gangoiti scale were analyzed with a sample in which the data collection was conducted in the presence of both parents (55.8%), in the presence of the mother (42.2%) and or the presence of the father (2%). In the case of HOME, almost all the data were collected with mothers. Notably, HOME and Etxadi-Gangoiti are intended to assess the general characteristics of the familial environment as a developmental context for the child. Interviews and questionnaires are the primary methods to gain information, and direct observation is a specific strategy to obtain information about some aspects.

Parents' reports can offer valuable information about parental practices from the parent's perspective, especially to assess their attitudes and knowledge about parenting and child development. However, research has shown that parent self-reports can differ from actual practices. Therefore, direct observation of adults' behaviors when they interact with their sons and daughters can provide different, complementary information (Comfort et al., 2011). Parental reports about their own behaviors can be biased by their interpretation of the items or by a desirability bias. Direct observation may allow us to collect more accurate, valid data about actual parental behaviors (Comfort et al., 2011; Roggman et al., 2013a).

As the aim is not to conduct an exhaustive, systematic review, the Keys to Interactive Parenting Scale (KIPS) can be taken as an example of an observational tool to assess parental behaviors (Comfort et al., 2011). It was validated in the United States with a sample including 94% of mothers. Mention should also be made of the Maternal Behavior Rating Scale-Revised (MBRS; Mahoney, 2008), a tool which has been widely applied to mothers and has also been used with fathers (Van Keer et al., 2017). Interestingly, in a systematic review conducted by Lotzin et al. (2015), only one of 24 observational measures of parent-infant interactions included fathers in the validation study.

In Spain, Trenado et al. (2014) developed the reviewed version of the Early Mother–Child Interaction Coding System (CITMI-R), an observational coding system to analyze mother–child interactions with babies. The authors stated that the tool could be used to analyze fathers' or other caregivers' interactions with babies, but it was initially developed and almost exclusively used with mothers. This tool is time-intensive to code. It analyzes mother–child interactions in the first year of the baby's life and it has not been broadly used cross-culturally.

PICCOLO is an observational tool that has proved easy to use, with high levels of reliability and validity. It is suitable for different ethnic and cultural groups and nationalities, applicable to assess adult–child interactions from 10 to 47 months, and can be used to analyze interactions in the natural contexts in which they occur (Roggman et al., 2013a,b). It is composed of 29 items on parenting behaviors in four behavioral domains (Affection, Responsiveness, Encouragement, and Teaching). Each domain refers to specific kinds of developmentally supportive parenting behaviors that predict children's developmental outcomes (Innocenti et al., 2013; Roggman et al., 2013a,b).

PICCOLO was initially developed in the United States, following an in-depth review of the literature about parental behaviors related to positive children's developmental outcomes and the authors' own studies with a sample of 2,048 mothers from different ethnic groups. The psychometric properties of the Turkish and Spanish versions of PICCOLO have also been analyzed, with samples of 156 and 203 mothers respectively (Bayoglu et al., 2013; Vilaseca et al., 2019b).

PICCOLO's main authors considered that it could not be assumed that the same tool could be used to assess fathers' parental interactions (Anderson et al., 2013). It was necessary to check whether behaviors that have been shown to promote child development coincide in mothers and fathers or whether some behaviors should be excluded or added when fathers' interactions with their children are assessed. To develop PICCOLO-D (Anderson et al., 2013), the fathers' version of PICCOLO, the authors tested the psychometric properties of the 29 original PICCOLO items and 44 additional ones that were identified from a review of the literature on early fathering. The results showed that it was advisable to validate the tool for its use with fathers. Because of the psychometric process, none of the additional items were included in PICCOLO-D and only 21 of the 29 original PICCOLO items met the criteria.

Based on this context, we considered the advisability of assessing whether the Spanish version of PICCOLO (Vilaseca et al., 2019b) accomplished psychometric standards for use with fathers. In Spain, PICCOLO has been used in research with families that have children with intellectual disabilities (Vilaseca et al., 2019a, 2020) and with families that have typically developing children (Rivero et al., under review)^1^. It is also beginning to be used in clinical intervention contexts, mainly in early intervention centers, to improve parental behaviors in international proposals (Roggman et al., 2020) and in Spain (Vilaseca and Pastor, 2019; Portilla et al., 2021). Professionals have reported than PICCOLO is an easy-to-administer and easy-to-score observational tool that can provide accurate data about parental behaviors and is sensitive to changes in response to intervention. As mentioned above, the literature is growing on the contributions of fathers to child development. Therefore, fathers must be included in early intervention programs that follow capacity-building family-system intervention practices (Dunst et al., 2019). Undoubtedly, having reliable, valid tools to assess fathers' parental behaviors for professional and research purposes is highly useful and necessary.

Therefore, the aim of the current study was to test the psychometric properties of PICCOLO in a sample of 191 Spanish fathers in interactions with their children aged from 10 to 47 months, to check whether the same tool and the same test standards could be used for Spanish mothers and fathers or whether some items should be removed, specific standards should be drawn up, or another tool would be needed to assess fathers' parenting. We hypothesize that the Spanish version of PICCOLO (Vilaseca et al., 2019b) will meet the psychometric criteria found in previous studies with mothers.

## Materials and Methods

### Participants

The sample included 191 father–child dyads who were video-recorded playing together. They were recruited from pediatric centers, nurseries, and community family centers. The inclusion criteria were: (a) child's age between 10 and 47 months; (b) normal weight and no complications in childbirth, and (c) no hospitalizations prior to enrollment in the study. The fathers who participated in this study were from the same families as the mothers who participated in the validation of the Spanish version of PICCOLO (Vilaseca et al., 2019b). Mothers and fathers interacted with the same son or daughter.

Regarding the children, 55.0% were male (45.0% female), aged from 10 to 47 months (*M* = 27.97, *SD* = 9.07). More specifically, 16.6% of children were <1 year old (10–11 months), 33.5% were 1 year old (12–23 months), 43.5% were 2 years old (24–35 months), and 21.5% were 3 years old (36–47 months). Fathers were aged between 25 and 53 years (*M* = 37.31, *SD* = 5.31). Most of the fathers were married or living with a partner (99.5%). A total of 54.5% had a university degree, 36.8% had completed high school or a vocational training program, and 8.7% had received only elementary schooling. They were either fully employed (95.1%), partially employed (3.3%) or unemployed (1.6%). A total of 27.1% of the sample had a monthly family net income between €1,313 and €2,451, which is considered an average income in Spain. Of the families, 2.8% had a monthly income below €1,313, and the remaining families (70.2%) had a monthly income above €2,451.

### Instruments

The Spanish version of PICCOLO (Vilaseca et al., 2019b) includes the 29 observable behaviors of the original tool (Roggman et al., 2013a,b). It has been shown to meet psychometric criteria with a sample of 203 mothers interacting with their children, aged 10–47 months.

The 29 items refer to parental behaviors that have proved to predict the child's developmental outcomes. Every item is scored according to its frequency and consistency as 0 (absent; no behavior observed), 1 (barely; minor or emerging behavior) and 2 (clearly; definitive, strong, frequent behavior). The items are grouped into four domains: (a) Affection (seven items), which involves physical and verbal expression of affection, positive emotions, positive evaluation and positive regard; (b) Responsiveness (seven items), which refers to being attentive to the child's signals, interpreting and responding to them in a suitable, contingent way, following their interests and needs; (c) Encouragement (seven items), which refers to non-intrusive parental control and the parents' support of children's efforts, exploration, autonomy, choices, creativity and initiative; and (d) Teaching (eight items), which includes cognitive, conversational and linguistic stimulation behaviors. The instrument generates a score for each dimension between 0 and 14 (and 0 to 16 for the Teaching dimension) and a total score between 0 and 58 (by summing all the items). The Spanish version of PICCOLO used to observe mothers interacting with children was found to fit the original factor structure (i.e., four first-order factors and one second-order factor) and to provide reliable scores in terms of inter-rater reliability (intra-class correlation coefficient of 0.84 for total scores, and 0.83 for Affection,0.69 for Responsiveness,0.81 for Encouragement and 0.80 for Teaching), and internal consistency (Cronbach's alpha for total score was 0.88, and 0.59 for Affection, 0.75 for Responsiveness, 0.79 for Encouragement and 0.68 for Teaching).

The Spanish version of the Bayley Scales of Infant Development—III (BSID-III; Bayley, 2015) was used to assess the child's development. Cognitive, Expressive Language, Receptive Language, Fine Motor and Gross Motor subscales were applied. Bayley-III has demonstrated high reliability and validity in Spain (Bayley, 2015; Castro and Cobos, 2017).

### Procedure

Ethical approval was obtained from the University of Barcelona's Bioethics Commission (CBUB), according to the International Ethical Guidelines for Health-related Research Involving Humans prepared by the Council for International Organizations of Medical Sciences (CIOMS) in collaboration with the World Health Organization (WHO), and the WMA Declaration of Helsinki—Ethical Principles for Medical Research Involving Human Subjects.

Pediatric centers, nurseries, and community family centers were contacted by letter and telephone. Professionals from the centers were informed about the study, and they were asked to collaborate in recruiting participants. The parents were informed that their participation would be entirely voluntary and anonymous. Information about the study, informed consent, a demographic questionnaire, and a brief guide about how to video-record adult–child interaction during play at home were delivered to the parents.

Fathers were asked to video-record at home a free-play session with their son or daughter, for ~10 min (between 8 and 10 min), according to the following instruction: “Interact and play with your children as you typically do.” Some activities that elicit interaction and communication were suggested (books, toy animals, kitchens, little dolls, building blocks, etc.). The toys selected by fathers were very similar to those selected by mothers in the previous study of Spanish validation of PICCOLO (Vilaseca et al., 2019b). Additionally, fathers were given the following written instructions for carrying out the recording: play in a quiet place; play alone with the child, without the presence of other people; avoid ambient noises; have good lighting conditions; and frame the interaction to clearly see gestures, facial expressions and the use of play materials, focusing the camera in a way that also captured the mobility that might occur during play. All instructions given were in accordance with the PICCOLO manual (Roggman et al., 2013b).

Videos that met the given instructions (between 8 and 10 min) and were clear in sound and image (95%) were scored according to PICCOLO by a university research group. All scorers were psychologists and specialists in child development. The first author, who was trained by the authors of the original PICCOLO, trained the raters. Observer trainees read about the content and purpose of the measure (during a 3-h session) and watched and discussed four video recordings (3 h). At the end of the training sessions, the observers watched and coded 4–6 additional video-recorded interactions to establish reliability (3–6 h). The observers were considered to have completed their training satisfactorily when the percentage of inter-rater agreement was equal to or above 80%.

To test criterion-related validity, a subsample of 82 children were randomly selected and assessed using the cognitive, linguistic and motor subscales of the Bayley Scales of Infant Development (BSID-III; Bayley, 2015). The smaller number of participants for this subsample was due to the high cost of applying the Bayley Scales to the children.

### Data Analysis

Inter-rater reliability was estimated through two coders who independently rated 32 video observations. Specifically, the percentage of agreement for each item and the intraclass correlation coefficient (ICC; two-way mixed effects, absolute agreement) were obtained for each PICCOLO domain and total score.

Validity and internal consistency evidence of PICCOLO scores were gathered from video observations rated by one of the two coders (*n* = 191). Confirmatory factor analysis (CFA) was used to test the data fit to the dimensional structure of the original version of the scale: four first-order factors (Affection, Responsiveness, Encouragement, and Teaching) and one second-order factor (Parenting). Due to the ordered categorical nature of the data, the diagonally weighted least squares (DWLS) estimator based on a polychoric correlation matrix was applied, since this model is considered a robust estimator for ordinal data, small samples, and violations of normality (Forero et al., 2009). The model fit was assessed with the CFI, the TLI and the RMSEA [90% CI], following standard guidelines that suggest values of CFI ≥ 0.95, TLI ≥ 0.95, and RMSEA ≤ 0.06 as indicating a good fit, and CFI ≥ 0.90, TLI ≥ 0.90, and RMSEA ≤ 0.08 as indicating a reasonable fit (Hu and Bentler, 1999; Marsh et al., 2005). Internal consistency was assessed by means of ordinal alpha (α) and McDonald's omega (ω), both based on the polychoric correlation matrix. Pearson's correlation coefficient was computed to analyze the relationship between age (in months) and PICCOLO scores, and an ANOVA was used to compare scores between age groups (1, 2, and 3 years). Mean PICCOLO scores were also compared between boys and girls by means of Student's *t*-test. Scores on PICCOLO's domains and total score were correlated with each other and with the BSID-III (scalar scores on cognitive, language and motor skills) by means of Pearson's correlation coefficient.

The R packages *lavaan* (Rosseel, 2012) and *semTools* (Jorgensen et al., 2019) were used, respectively, for the CFA and the internal consistency analyses.

## Results

### Inter-rater Reliability

The 32 video observations (16.8%) that were independently rated by two coders were used to estimate inter-rater reliability. The percentage of inter-coder agreement at item level ranged from 62.5 to 93.8%. More specifically, the items with the lowest and highest percentages of agreement in each domain were respectively: (a) Affection (item 2 *Smiles at child*, item 5 *Uses positive expressions with child*, item 6 *Is engaged in interacting with child* [78.1% each], and item 4 *Is physically close to the child* [90.6%]); (b) Responsiveness (item 2 *Changes pace or activity to meet child's interests or needs* [71.9%], and item 1 *Pays attention to what child is doing* [90.6%]); (c) Encouragement (item 6 *Offers suggestions to help child* [71.9%], and item 2 *Encourages child to handle toys* [93.8%]); and (d) Teaching (item 6 *Does activities in a sequence of steps* [62.5%], and item 4 *Label objects or actions for child* [90.6%]). Averaging these percentages across items within domains gave values of 82.1% for the Affection domain, 81.3% for the Responsiveness domain, 79.9% for the Encouragement domain, 78.1% for the Teaching domain, and 80.3% for all the items of the Spanish PICCOLO. The ICCs for each domain and total scores were also obtained, resulting in coefficients of 0.85 for Affection, 0.89 for Responsiveness,0.90 for Encouragement, and 0.93 for Teaching. For the total Spanish PICCOLO score, the ICC was 0.93. Altogether, these results show that the agreement between the two raters in scoring each PICCOLO item was high, resulting in high inter-rater reliability at the domain and total score level.

### Item Descriptive Statistics

Table 1 shows the percentage of endorsement of each item response category, and the item central tendency and dispersion measures. Responses were distributed along item response categories in most of the PICCOLO items, but most item endorsements were located at the upper level of the response scale. Teaching was the domain with the lowest mean item scores.

**Table 1:** Descriptive statistics of PICCOLO items.

	**Percentage of endorsement**				
**Domain/item**	**0**	**1**	**2**	**Mean**	**SD**
**Affection**					
(1). Habla con un tono de voz cariñoso [Speaks in a warm tone of voice]	0.0	12.0	88.0	1.88	0.33
(2). Sonríe al/a la niño/a [Smiles at child]	6.8	27.7	65.4	1.59	0.62
(3). Elogia al/a la niño/a [Praises child]	9.9	28.8	61.3	1.51	0.67
(4). Está físicamente cerca del/de la niño/a [Is physically close to the child]	0.5	11.0	88.5	1.88	0.34
(5). Utiliza expresiones positivas con el/la niño/a [Uses positive expressions with child]	62.3	17.8	19.9	0.58	0.80
(6). Se implica plenamente en la interacción con el/la niño/a [Is engaged in interacting with child]	0.5	12.0	87.4	1.87	0.35
(7). Muestra calidez emocional [Shows emotional warmth]	2.6	18.8	78.5	1.76	0.49
**Responsiveness**					
(1). Presta atención a lo que hace el/la niño/a [Pays attention to what child is doing]	0.0	14.1	85.9	1.86	0.35
(2). Cambia el ritmo o la actividad para ajustarse a los intereses o las necesidades del/de la niño/a [Changes pace or activity to meet child's interests or needs]	6.3	29.8	63.9	1.58	0.61
(3).Es flexible ante el cambio de actividades o intereses del/de la niño/a [Is flexible about child's change of activities or interests]	5.8	31.9	62.3	1.57	0.60
(4). Sigue lo que el/la niño/a está intentando hacer [Follows what child is trying to do]	1.0	20.4	78.5	1.77	0.44
(5). Responde a las emociones del/de la niño/a [Respond to child's emotions]	4.7	34.0	61.3	1.57	0.59
(6). Mira al/a la niño/a cuando este/a habla o emite sonidos [Looks at child when child talks or makes sounds]	5.2	17.3	77.5	1.72	0.55
(7). Responde a las palabras o los sonidos del/de la niño/a [Replies to child's words or sounds]	2.6	18.8	78.5	1.76	0.49
**Encouragement**					
(1). Espera la respuesta del/de la niño/a tras hacer una sugerencia [Waits for child's response after making a suggestion]	1.6	35.6	62.8	1.61	0.52
(2). Anima al/la niño/a a manipular juguetes [Encourages child to handle toys]	5.2	21.5	73.3	1.68	0.57
(3). Apoya al/a la niño/a para que tome la iniciativa [Supports child in making choices]	9.4	37.7	52.9	1.43	0.66
(4). Apoya al/a la niño/a cuando hace cosas por sí mismo/a [Supports child in doing things on his or her own]	4.7	35.6	59.7	1.55	0.59
(5). Anima verbalmente los esfuerzos del/de la niño/a [Verbally encourages child's efforts]	19.9	36.6	43.5	1.24	0.76
(6). Ofrece sugerencias para ayudar al/a la niño/a [Offers suggestions to help child]	17.8	34.0	48.2	1.30	0.76
(7). Muestra entusiasmo acerca de lo que está haciendo el/la niño/a [Shows enthusiasm about what child is doing]	4.2	27.2	68.6	1.64	0.56
**Teaching**					
(1). Explica al/a la niño/a las razones acerca de algo [Explains reasons for something to child]	47.1	22.5	30.4	0.83	0.87
(2). Sugiere actividades para ampliar lo que el/la niño/a está haciendo [Suggests activities to extend what child is doing]	18.3	30.9	50.8	1.32	0.77
(3). Repite o expande las palabras o los sonidos del/de la niño/a [Repeats or expands child's words or sounds]	7.3	37.2	55.5	1.48	0.63
(4). Da nombre a objetos o acciones [Label objects or actions for child]	5.2	25.1	69.6	1.64	0.58
(5). Se implica con el/la niño/a en juego de ficción [Engages in pretend play with child]	43.5	13.6	42.9	0.99	0.93
(6). Realiza actividades en una secuencia de pasos [Does activities in a sequence of steps]	41.4	18.8	39.8	0.98	0.90
(7). Habla al/a la niño/a acerca de las características de los objetos [Talks to child about characteristics of objects]	26.2	32.5	41.4	1.15	0.81
(8). Pide información al/a la niño/a [Asks child for information]	6.3	22.5	71.2	1.65	0.60

### Dimensional Structure

**Figure 1:**
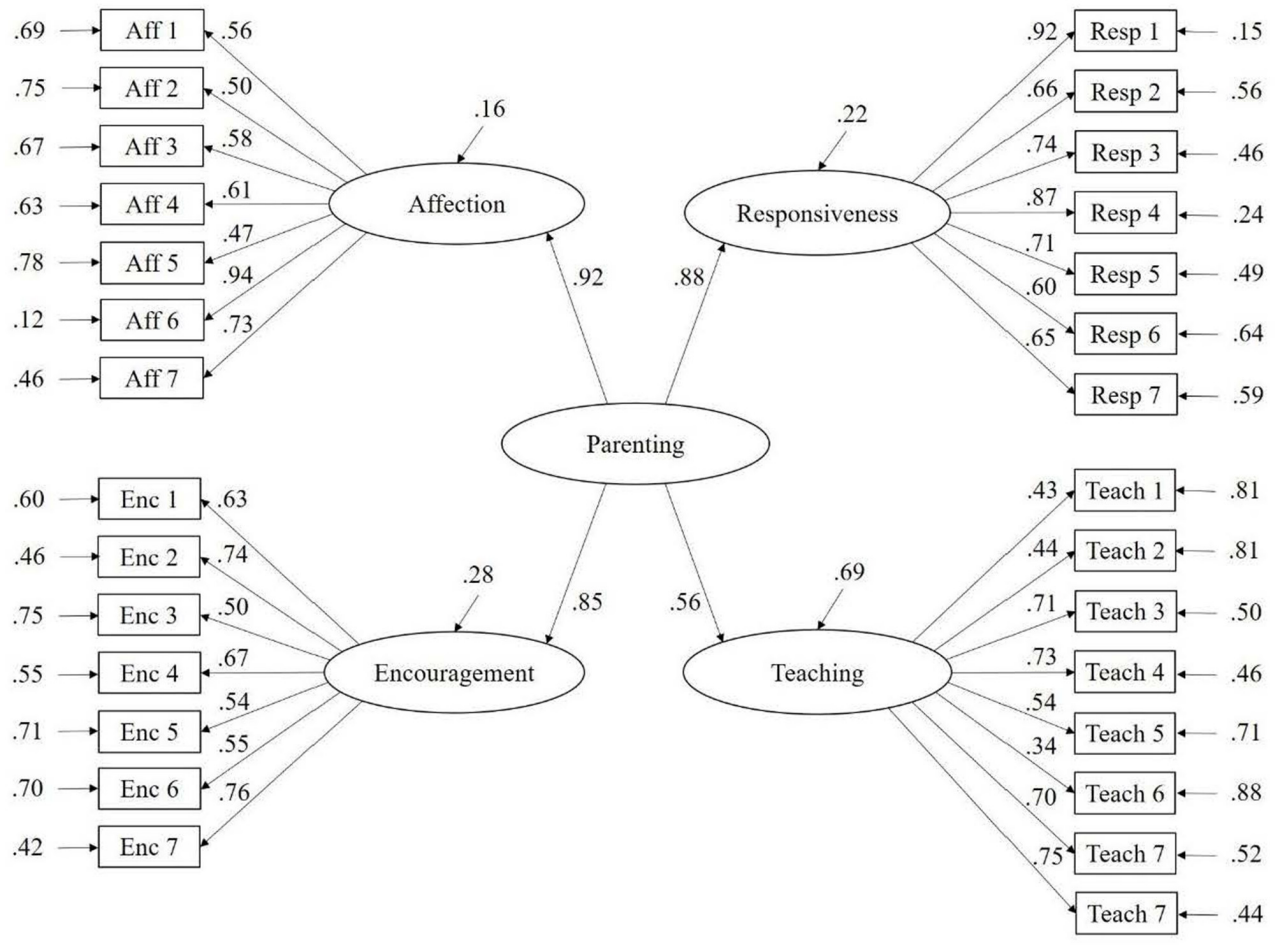
Path diagram of the original PICCOLO dimensional structure.

The model that was tested by CFA was based on the original PICCOLO dimensional structure (Roggman et al., 2013a), with four first-order factors corresponding to the four PICCOLO domains, and one second-order factor conforming a general factor of positive parenting interactions with children. The goodness of fit indices were CFI = 0.93, TLI = 0.92, and RMSEA [90% CI] = 0.070 [0.063–0.078], which suggests a reasonable fit. Most of the standardized residuals (95.1%) were in the recommended range (i.e., −2.58–2.58), which supports the fit of the model. Except for item 6 of the Teaching domain, factor loadings were high (λ > 0.40) and statistically significant, which suggests the items are adequate indicators of the corresponding latent variables (i.e., Affection, Responsiveness, Encouragement and Teaching). Figure 1 presents the path diagram of the CFA with items loading on one of the four domains and domains loading on the general factor.

### Internal Consistency

Video observations rated by one of the two coders (*n* = 191) were used to estimate the internal consistency of Spanish PICCOLO scores. Ordinal alpha and omega coefficients for each domain were as follows: Affection (α = 0.80, ω = 0.64), Responsiveness (α = 0.85, ω = 0.79), Encouragement (α = 0.81, ω = 0.73) and Teaching (α = 0.79, ω = 0.70). Based on these results, PICCOLO scores had adequate internal consistency.

### Relationships Between Domains

The Pearson correlation coefficients among Spanish PICCOLO domains indicated moderate relations between the Teaching and Affection domains (*r* = 0.34), Responsiveness (*r* = 0.32), and Encouragement (*r* = 0.30) domains, and high correlations between Responsiveness and Affection domains (*r* = 0.60) and between Encouragement and Affection (*r* = 0.51) and Responsiveness (*r* = 0.52) domains. Even though some of these correlation coefficients were substantial, none exceeded the value of 0.60. This suggests reasonable discriminant validity of the factors (Kline, 2015).

### Scores by Age and Gender

The mean and standard deviation for the Spanish PICCOLO scores according to the age of the child (children aged <1 year were excluded due to the small sample size; *n* = 3) are shown in Table 2. Pearson's correlation coefficients between child age (in months) and PICCOLO scores were obtained. Statistically significant correlations, albeit of low strength, were found between child age and scores for the Affection domain (*r* = −0.17; *p* < 0.05) and for the Teaching domain (*r* = 0.16, *p* < 0.05). ANOVA showed a main effect of age group for Teaching scores [*F*_(2,187)_ = 4.445; *p* = 0.013], and *post-hoc* analysis revealed that differences were between children with 1 vs. 2 years of age. No statistically significant differences were found between boys (*n* = 105) and girls (*n* = 86) in any PICCOLO domain or in the total score.

**Table 2:** PICCOLO scores by child age.

**Child age**	***n***	**Affection**	**Responsiveness**	**Encouragement**	**Teaching**	**Total**
		***M*** **(*SD*)**	***M*** **(*SD*)**	***M*** **(*SD*)**	***M*** **(*SD*)**	***M*** **(*SD*)**
1 year	64	11.27 (2.11)	11.67 (2.38)	10.19 (2.71)	9.19 (3.88)	42.31 (8.35)
2 years	83	11.25 (2.08)	12.14 (2.16)	10.84 (2.72)	10.84 (2.77)	45.08 (7.15)
3 years	41	10.41 (1.70)	11.34 (2.46)	10.39 (2.60)	10.10 (3.48)	42.24 (7.79)

### Relationship Between PICCOLO and BSID-III Scores

Pearson's correlations between fathers' PICCOLO scores and children's BSID-III scores were obtained (Table 3). Scalar scores of the BSID-III were used. Moderate positive correlations were found between the Encouragement domain and receptive language scores (*r* = 0.32), and between the Teaching domain and expressive language (*r* = 0.34) and composite language (*r* = 0.31) scores. The child's cognitive and motor skills were not associated with any father's parenting domain.

**Table 3:** Correlations between PICCOLO and BSID-III scores.

	**BSID-III score**							
	**Cognitive**		**Language**			**Motor skill**		
**PICCOLO score**	**Scalar** **(*n* = 81)**	**Composite (*n* = 81)**	**Receptive scalar (*n* = 82)**	**Expressive scalar (*n* = 81)**	**Composite** **(*n* = 81)**	**Fine scalar** **(*n* = 68)**	**Gross scalar** **(*n* = 68)**	**Composite (*n* = 68)**
Affection	0.11	0.11	0.10	0.07	0.085	−0.03	0.11	0.02
Responsiveness	0.13	0.14	0.10	0.06	0.084	−0.11	0.05	−0.01
Encouragement	0.25	0.26	0.32^*^	0.10	0.20	0.01	0.15	0.08
Teaching	0.04	0.06	0.18	0.34^*^	0.31^*^	−0.10	0.09	0.03
Total	0.18	0.20	0.25	0.22	0.25	−0.08	0.14	0.05

* *p < 0.01*.

## Discussion

The aim of our study was to test the psychometric properties of PICCOLO in a sample of Spanish fathers when they interact with their children at early ages, to check whether the tool can be used in its form for mothers, should be adapted for its use with fathers or even whether another tool should be developed. The fact that 8 of the 29 PICCOLO original items were excluded in the fathers' version of the PICCOLO in the United States (Anderson et al., 2013) indicated the advisability of checking whether the original PICCOLO was appropriate to assess Spanish fathers' parental behaviors, as was the case for Spanish mothers (Vilaseca et al., 2019b).

The results of the exploration of the psychometric properties of the PICCOLO with our sample indicate that the same tool can be applied to assess Spanish mothers' and fathers' parenting, as expected based on previous studies comparing the parental behaviors of Spanish mothers and fathers with children with intellectual disabilities (Vilaseca et al., 2020) and those with typically developing children (Rivero et al., under review)^1^.

Specifically, our study confirms the dimensional structure composed of four first-order factors and one second-order factor of parenting. The correlation coefficients among domains suggest reasonable discriminant validity of the factors. Except for item 6 of the Teaching domain, factor loadings were all high and statistically significant. Although this item does not meet the required psychometric requirements and is also the one with the lowest inter-rater agreement, we decided to retain it in the PICCOLO scale for several reasons: (a) the inter-rater reliability at Teaching domain level is adequate; (b) the item presents variability in scores; (c) the goodness of fit of the CFA is acceptable; (d) the internal consistency indexes of the Teaching domain are appropriate; and (e) maintaining the item enables comparisons between mothers and fathers within the Spanish context and potentially at cross-cultural level.

In this study, inter-rater agreement and internal consistency values are good and even higher than those of other studies of validation of the PICCOLO in the United States with mothers (Roggman et al., 2013a,b) and fathers (Anderson et al., 2013) or those carried out in other countries such as Turkey (Bayoglu et al., 2013) and Spain (Vilaseca et al., 2019b) with mothers.

With respect to the child's age, the mean scores in all the parenting domains were very similar at 1, 2, and 3 years. Teaching was the exception, which showed a significant increase between 1 and 2 years of the child's age. Fathers' Teaching behaviors were more common with older children, as was the case in the original PICCOLO sample (Roggman et al., 2013a,b), and the Turkish and the Spanish validation of PICCOLO with mothers (Bayoglu et al., 2013; Vilaseca et al., 2019b). The consistency in the results about Teaching indicates that these behaviors (e.g., explaining reasons for something to the child, talking about the characteristics of objects, asking the child for information, etc.) are clearly more frequent with older children. This result indicates that parents are adjusting their Teaching behaviors to their perception of their children's communicative, linguistic, and cognitive skills, and perform fewer Teaching behaviors with younger children. Parents of 1-year-old children would be more inclined to perform affective behaviors, to adjust to the child's interests and necessities, and to stimulate the child's growing autonomy of action, and less willing to perform Teaching behaviors related to stimulating language and cognition. Since the Teaching behaviors assessed with PICCOLO have shown to be related to the child's cognitive and linguistic development at early ages, both in typically developing children and in children with disabilities (Roggman et al., 2013a; Vilaseca et al., 2019a), the lesser presence of Teaching behaviors when interacting with children at early stages of development is particularly relevant for intervention programs with families with a child with developmental delays or disabilities, or a child at risk. Interestingly, research shows that parents may tend to underestimate their children's abilities (Chung et al., 2019), and may therefore desist from carrying out certain Teaching behaviors considered to be beyond them. Therefore, promoting and supporting parents' Teaching should receive special attention in early intervention and childcare programs.

In the fathers' sample, Affection slightly decreased with age. This negative correlation was not found in the Spanish validation of PICCOLO with mothers, but it was a result of the Turkish validation (Bayoglu et al., 2013). Therefore, according to these data, the mothers' and fathers' tendency to increase Teaching behaviors with older children appears to be a commonality in the countries in which the PICCOLO has been validated, while the tendency to diminish affective behaviors it is not so clearly a general tendency. More research is needed to establish whether this indicates cultural differences with respect to the expression of affection. With respect to other dimensions of parenting, Responsiveness and Encouragement tended to remain fairly constant at all ages, as was the case in the aforementioned studies. Fathers would adjust to the child's interests and needs to encourage the child's efforts and autonomy at all ages. No significant relation was found between any parenting domains or total parenting scores and the child's gender, as was also the case in the previous studies of validation of the PICCOLO.

Concurrent validity between PICCOLO and BSID-III showed fewer positive associations than expected according to the literature about fathers' parental behaviors and child development. Positive associations were found between the fathers' scores in Encouragement and the child's scores in receptive language, and between Teaching and expressive language and total language scores. Although relations between Encouragement and Teaching and the child's linguistic development were expected (Baker et al., 2015; Rowe et al., 2017; Reynolds et al., 2019; Vilaseca et al., 2019a), other documented relations were not found. The relations between fathers' Responsiveness and the child's linguistic development seems to be well-established in the literature (Tamis-LeMonda et al., 2013), but it was not found in our data. We also expected to find some relation between Encouragement and the child's cognitive development, as suggested from the studies of Meuwissen and Carlson (2015), and between Affection and linguistic development (McMahon et al., 2019). Compared to our previous validation of PICCOLO with Spanish mothers (Vilaseca et al., 2019b), all the associations identified in the fathers' study were also reported for mothers. Additionally, for mothers, Responsiveness and the total PICCOLO scores were associated with the child's language, and Encouragement was associated with the child's cognitive outcomes. In both studies, as expected for the kind of activities that were analyzed (book-reading, symbolic play, drawing, blocks, etc.), motor skills were not associated with any fathers' parenting domain.

In our study, only the child's linguistic outcomes appear to be related to fathers' teaching behaviors. Despite this, the relations between the parental domains that were explored and the child's linguistic, cognitive and socioemotional developmental outcomes are well-established (for reviews, see Roggman et al., 2013a,b; Fitzgerald et al., 2020). More research on fathers' parenting with young children in different countries and contexts is required to expand knowledge about its contribution to the child's development.

PICCOLO has been used to assess parenting interactions in Brazil, Chile, China, Germany, Italy, Spain and Turkey, mostly with mothers (personal communication with the PICCOLO authors), and it was originally developed and validated with a sample of multiple ethnic groups in the United States (Roggman et al., 2013a,b). Our study contributes to increasing the evidence that the tool is useful to capture a set of behaviors that are strongly linked to positive developmental outcomes in different populations and cultural contexts and with different caregivers. It is well-known that parental practices are diverse among cultures, and they reflect cultural beliefs and values about childrearing and child development (Bornstein et al., 2007; Brophy-Herb et al., 2012). However, many developmental milestones, parenting strategies and family processes are likely to be similar across cultures (Bornstein, 2013). This seems to be the case for PICCOLO items, as they refer to behaviors that can be specified in different ways according to cultural differences (e.g., referring to different objects and activities), but adjusting to the same general description and having the same meaning.

The Spanish validation of PICCOLO to assess mothers' and fathers' parental behaviors linked to developmental outcomes can be useful for further studies about the parents' contribution to a child's development. This is especially true for fathers, given the growing interest in the father's involvement in childrearing and the need to expand research on this topic to different countries and populations. It is particularly interesting to develop and validate observational tools for parenting research, given that in this area there is a predominance of parental reports. As we mentioned in the introduction, parents' reports can offer valuable information about parental practices from the parents' perspective but may differ from actual practices (Comfort et al., 2011). Direct observation is of special interest to collect accurate, valid data about actual parental behaviors (Comfort et al., 2011; Roggman et al., 2013a). The ease of use and the little time required for administration are advantages of PICCOLO in comparison to other observational tools that were reviewed and mentioned in the introduction.

Another relevant advantage of PICCOLO is that it is an assessment tool linked to intervention goals. This is of particular interest for professional practice and applied research. Family programs and early intervention practitioners usually include as a relevant aim of their interventions to improve parenting skills and behaviors that promote the child's development (Vilaseca and Pastor, 2019; Roggman et al., 2020; Portilla et al., 2021). Assessment tools that are clearly linked to intervention goals, as is the case of PICCOLO, are of particular interest for practitioners. The 29 items of PICCOLO are not only items for the assessment of parental strengths prior to an intervention, but a guide that can be used throughout the intervention process to establish strategies and goals and reflect together with parents about parental competencies. The growing interest in implementing evidence-based practices has led to an increasing need for reliable and valid tools for assessment and intervention. The Spanish version of PICCOLO, which has proved reliable and valid to assess mothers' and fathers' parental behaviors, could be of particular interest for practitioners in our country, especially those working from collaborative models with parents, including mothers and fathers, and a family-centered approach based on parental competencies and daily-routines (McWilliam, 2010, 2016; Mas et al., 2016; Vilaseca et al., 2017). As parental skills are not necessarily the same for both members of the couple, and to some extent can compensate for each other within a family (Cabrera et al., 2014), assessing mothers' and fathers' parenting behaviors and incorporating and integrating the strengths of each of the parents may well-benefit the child's development. This idea can be extended to families with two mothers or two fathers.

Beyond their contributions, our study has some limitations to consider, and new directions of research could be of interest.

First, our study was conducted with a non-probabilistic sample, with a predominance of fathers who had completed university studies and had family incomes above the average in Spain. Future studies with broader samples including fathers from different socioeconomical backgrounds could be of interest to reinforce the validation of the tool. Gathering data from other Spanish speaking countries could also be of interest.

Furthermore, although Bayley-III is a reliable and valid tool to assess the child's development during the first three-and-a-half years of age, further studies with other measures of the child's developmental outcomes should be developed, to gather new data about the concurrent validity of PICCOLO and the child's development. Particularly, it would be of interest to include an assessment of the child's socioemotional development, which is not considered in our study, with the Spanish version of ASQ-3 (Squires and Bricker, 2009) or other instruments.

Finally, construct validity of the tool in the Spanish population should be explored, to relate PICCOLO scores with those of other tools designed to assess parenting that have been validated in Spain. Examples are the CITMI-R (Trenado et al., 2014), the Questionnaire for the Early Assessment of Parental Competencies (Vázquez et al., 2016) or the Spanish form of KIPS (Comfort et al., 2011).

Beyond these limitations, our study makes a relevant contribution to the field of parental interactions and child development, enriching the set of instruments validated in the Spanish population that can be used in research, to continue deepening the study of parental behaviors related to the child's outcomes, and in professional contexts, to improve adult–child dyadic interactions and the child's development. There are few measures to capture the quality of father–child interactions with their children in natural contexts, and this is a contribution of our work to the field. Furthermore, our study suggests that parental behaviors of mothers and fathers can be assessed with the same tools, without the need to develop specific instruments for fathers. This was not totally unexpected, as it is well-established that in Western cultures mothers' and fathers' parenting behaviors present more commonalities than differences (Cabrera et al., 2014). Some studies also indicate that mothers' and fathers' parental behaviors may grow more similar over time because of cohabitation (Osnat and Bonnie, 1995), probably because they rely on each other in searching for successful parental strategies (Schoppe-Sullivan et al., 2007).

Future studies with PICCOLO should help to broaden our knowledge of fathers' parenting behaviors as a function of variables such as educational level, income, degree of parental involvement in childrearing, or fathers' knowledge and ideas about child development and learning. Future research could also compare fathers' parenting when interacting in a dyadic way with their child and when they do so in triadic interactions (mother-father-child). PICCOLO could also be used to observe fathers' behaviors in structured tasks, in contrast to free-play, since recent research has highlighted the effects of context on parental behaviors (Vallotton et al., 2020).

## Conclusion

The Spanish version of PICCOLO, which was originally validated with a sample of mothers (Vilaseca et al., 2019b), meets psychometric criteria to assess fathers' parenting interactions. It is not necessary to make significant changes to the original PICCOLO or to develop a specific tool.

To have a validated tool that can be used to assess, by observation, both mothers' and fathers' parental behaviors is of interest for research and intervention purposes. As PICCOLO is an assessment tool that is clearly linked to intervention goals, it is of particular interest for practitioners of early intervention and family programs in Spain, especially for those working from collaborative models with parents, including mothers and fathers, and a family-centered approach based on parental competencies and daily routines (McWilliam, 2010, 2016; Mas et al., 2016; Vilaseca et al., 2017).

The relation between parental behaviors included in PICCOLO and the child's development is well-established in the literature, and a significant relation between the fathers' PICCOLO scores and the child's linguistic outcomes has been found in our study. Nevertheless, more research is needed to expand knowledge about the father's contribution to the child's development.

## Data Availability Statement

The datasets presented in this article are not readily available because the parents allowed our research team to video record their interaction with their children, but not to publish any personal data or image. Questions regarding the datasets should be directed to the authors.

## Ethics Statement

The studies involving human participants were reviewed and approved by University of Barcelona's Bioethics Commission (CBUB). Written informed consent to participate in this study was provided by the parents.

## Author Contributions

MR and RV conceived and designed the research. MR, RV, and FF collected the data. GG made the statistical treatment of the data. All authors interpreted the results, participated in drafting the article, revised it critically for important intellectual content, and gave final approval of the version to be submitted.

## Funding

This research was supported by a grant from the Spanish Ministry of Economy and Competitiveness and the European Regional Development Fund (Project PSI2015-63627-R) and the ARE2020.2 program of the Faculty of Education of the University of Barcelona. The funding bodies have not imposed any restrictions on free access to or publication of the research data.

## Conflict of Interest

The authors declare that the research was conducted in the absence of any commercial or financial relationships that could be construed as a potential conflict of interest.

## Publisher's Note

All claims expressed in this article are solely those of the authors and do not necessarily represent those of their affiliated organizations, or those of the publisher, the editors and the reviewers. Any product that may be evaluated in this article, or claim that may be made by its manufacturer, is not guaranteed or endorsed by the publisher.

## Footnotes

^1^ Rivero, M., Vilaseca, R., Cantero, M. J., Navarro-Pardo, E., Ferrer, F., Valls-Vidal, C., et al. (under review). Parenting of Spanish mothers and fathers playing with their children at home.
